# Exploring the ATR-CHK1 pathway in the response of doxorubicin-induced DNA damages in acute lymphoblastic leukemia cells

**DOI:** 10.1007/s10565-021-09640-x

**Published:** 2021-09-14

**Authors:** Andrea Ghelli Luserna Di Rorà, Martina Ghetti, Lorenzo Ledda, Anna Ferrari, Matteo Bocconcelli, Antonella Padella, Roberta Napolitano, Maria Chiara Fontana, Chiara Liverani, Enrica Imbrogno, Maria Teresa Bochicchio, Matteo Paganelli, Valentina Robustelli, Seydou Sanogo, Claudio Cerchione, Monica Fumagalli, Michela Rondoni, Annalisa Imovilli, Gerardo Musuraca, Giovanni Martinelli, Giorgia Simonetti

**Affiliations:** 1 Biosciences Laboratory, IRCCS Istituto Romagnolo per lo Studio dei Tumori (IRST) “Dino Amadori”, Via Piero Maroncelli, 40, 47014 Meldola, FC Italy; 2 Department of Experimental, Diagnostic and Specialty Medicine, Institute of Hematology “L. e A. Seràgnoli”, University of Bologna, Bologna, Italy; 3 Hematology Unit, IRCCS Istituto Romagnolo per lo Studio dei Tumori (IRST) “Dino Amadori”, Meldola, FC Italy; 4 Hematology Division and Bone Marrow Transplantation Unit, San Gerardo Hospital, Monza, Italy; 5 Hematology Unit, Ospedale Santa Maria delle Croci, Ravenna, Italy; 6 Hematology, AUSL-IRCCS di Reggio Emilia, Reggio Emilia, Italy; 7 Scientific Directorate, IRCCS Istituto Romagnolo per lo Studio dei Tumori (IRST) “Dino Amadori”, Meldola, FC Italy

**Keywords:** Doxorubicin, Cell cycle, ATR, CHK1, Acute lymphoblastic leukemia

## Abstract

Doxorubicin (Dox) is one of the most commonly used anthracyclines for the treatment of solid and hematological tumors such as B−/T cell acute lymphoblastic leukemia (ALL). Dox compromises topoisomerase II enzyme functionality, thus inducing structural damages during DNA replication and causes direct damages intercalating into DNA double helix. Eukaryotic cells respond to DNA damages by activating the ATM-CHK2 and/or ATR-CHK1 pathway, whose function is to regulate cell cycle progression, to promote damage repair, and to control apoptosis. We evaluated the efficacy of a new drug schedule combining Dox and specific ATR (VE-821) or CHK1 (prexasertib, PX) inhibitors in the treatment of human B−/T cell precursor ALL cell lines and primary ALL leukemic cells. We found that ALL cell lines respond to Dox activating the G2/M cell cycle checkpoint. Exposure of Dox-pretreated ALL cell lines to VE-821 or PX enhanced Dox cytotoxic effect. This phenomenon was associated with the abrogation of the G2/M cell cycle checkpoint with changes in the expression pCDK1 and cyclin B1, and cell entry in mitosis, followed by the induction of apoptosis. Indeed, the inhibition of the G2/M checkpoint led to a significant increment of normal and aberrant mitotic cells, including those showing tripolar spindles, metaphases with lagging chromosomes, and massive chromosomes fragmentation. In conclusion, we found that the ATR-CHK1 pathway is involved in the response to Dox-induced DNA damages and we demonstrated that our new in vitro drug schedule that combines Dox followed by ATR/CHK1 inhibitors can increase Dox cytotoxicity against ALL cells, while using lower drug doses.

**Graphical abstract:**

• Doxorubicin activates the G2/M cell cycle checkpoint in acute lymphoblastic leukemia (ALL) cells.

• ALL cells respond to doxorubicin-induced DNA damages by activating the ATR-CHK1 pathway.

• The inhibition of the ATR-CHK1 pathway synergizes with doxorubicin in the induction of cytotoxicity in ALL cells.

• The inhibition of ATR-CHK1 pathway induces aberrant chromosome segregation and mitotic spindle defects in doxorubicin-pretreated ALL cells.

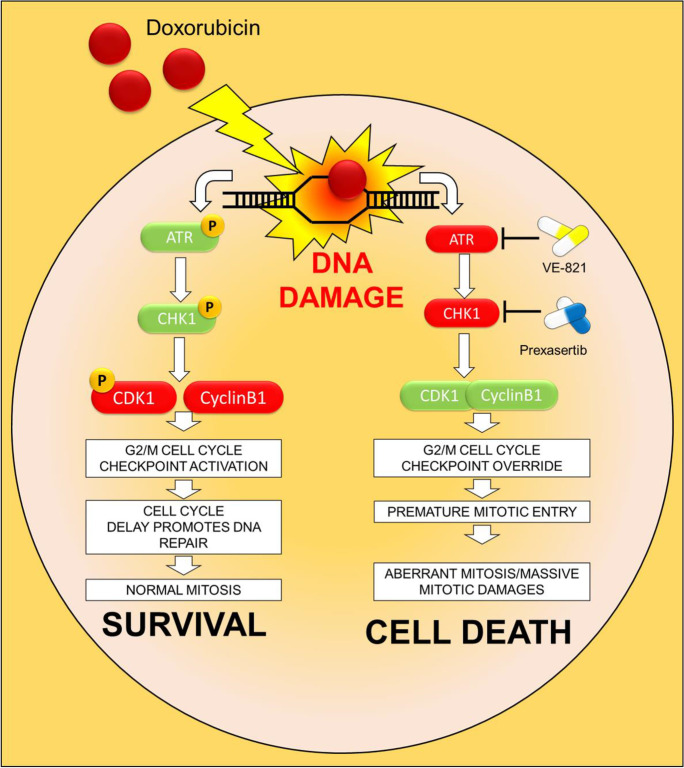

**Supplementary Information:**

The online version contains supplementary material available at 10.1007/s10565-021-09640-x.

## Background

Among the different types of DNA damages generated by endogenous or exogenous sources, those affecting single or double strands of DNA structure are the most deleterious ones for eukaryotic cells. Unrepaired single-strand (SSB) or double-strand breaks (DSBs) can compromise chromosome integrity leading to genetic instability (Cannan and Pederson 2016). Several tumor suppressors, involved in the DNA damage response (DDR) pathways, play a specific role in the identification and repair of these types of damages (Lupertz et al. 2010; Spina et al. 2013). Two groups of proteins, with diverse functionality, participate to the DDR pathways: (i) the cell cycle checkpoint-related kinases, which are involved in the initial steps of the response and promote cell cycle delay upon the identification of a DNA damage and (ii) the DNA repair proteins, which are involved in the resolution of the identified damage (Ghelli Luserna Di Rorà et al. 2017). Ataxia-telangiectasia mutated (ATM), ataxia- and Rad3-related (ATR) kinases and their downstream effectors (checkpoint kinase 2 (CHK2) and 1 (CHK1) kinase, respectively) play a central role in initial steps of the DDR pathways. Briefly, ATM-CHK2 kinases are active in response to DSBs while SSBs and the replicative stress (e.g., stalled replicative forks) trigger the ATR-CHK1 cascade (Blackford and Jackson 2017). Although DDR pathways act as tumor suppressors, altered expression (mainly over-expression) and inactivating mutations are frequent events in cancer cells and have been linked to chemoresistance (David et al. 2016; Meyer et al. 2020; Zhou et al. 2020). Doxorubicin (Dox) is an anthracycline used in the treatment of solid and hematological malignancies (Garcia-Manero and Kantarjian 2000; Kantarjian et al. 2000; Morris et al. 2011). Dox compromises topoisomerase II-mediated repair of supercoiled DNA structures and, consequently, induces severe DNA damages and, in particular, DSBs (Swift et al. 2006; Yang et al. 2014). Moreover, Dox cytotoxicity has been associated with the generation of free oxygen radicals (ROS) and the induction of oxidative stress (Navarro et al. 2006; Thorn et al. 2011). Despite being effective against highly proliferating cells, Dox has severe side effects (long-term cardiotoxicity and nephrotoxicity) that prevented its extensive use in the clinics (Lipshultz et al. 2004; Minotti et al. 2004). Dox is a component of the hyperfractionated cyclophosphamide, vincristine, doxorubicin, and dexamethasone (Hyper-Cvad) regimen that is currently used in the treatment of acute lymphoblastic leukemia (ALL) (Garcia-Manero and Kantarjian 2000; Kantarjian et al. 2000; Morris et al. 2011). Despite an initial overall response to chemotherapy agents (85–92% of cases (Terwilliger and Abdul-Hay 2017)), a large percentage of ALL patients relapse or become refractory to conventional therapies (Ganzel et al. 2020). Beside the known mechanisms of chemoresistance, including the downregulation of Dox targets (e.g., TOP2A (Harker et al. 1995)) and the upregulation of drug transporters (e.g., ABCC1 (Berrazouane et al. 2019)), an excessive DDR kinase activity has been demonstrated to play a central role in drug cytotoxicity (Salunkhe et al. 2018; Stefanski et al. 2019). Different studies showed that Dox activates cell cycle checkpoints and induces DNA damages (Kurz et al. 2004; Morii et al. 2015; Shin et al. 2015; Takeuchi et al. 2019; Vera et al. 2015). DDR inhibition by selective targeting of ATM (Batey et al. 2013), DNA-PK (Davidson et al. 2012), CHK1/CHK2 (Baranski et al. 2015; Chung et al. 2018; Weng et al. 2015), WEE1 (Aarts et al. 2012; Alikarami et al. 2017; Bridges et al. 2011; Ghelli Luserna Di Rorà et al. 2018; Hirai et al. 2009; Rajeshkumar et al. 2011), and PARP-1 (Park et al. 2018) enhances Dox cytotoxicity against hematological and solid tumor models.

In the present study, we aimed to design a drug schedule to make ALL cells dependent from the functionality of the G2/M checkpoint and to combine selective cell cycle checkpoint inhibitors to override the block and to promote cell death. We found that ALL cells exposed to Dox activate primarily the G2/M phase checkpoint and that the inhibition of the ATR-CHK1 pathway promotes cell cycle checkpoint override and premature mitotic entry, ending in cell death.

## Material and methods

### Cell lines

Three ALL cell models have been chosen as representative of the main ALL sub-type and, in particular, T cell ALL (RPMI-8402), Philadelphia-positive B cell ALL (SUP-B15), and Philadelphia-negative B cell ALL (REH). All the cell lines were obtained from Leibniz-Institut DSMZ-Deutsche Sammlung von Mikroorganismen und Zellkulturen GmbH (Germany) and were cultured according to manufacturer’s instructions.

### Primary leukemic cells

Primary samples were collected after obtaining written informed consent. The study was approved by Comitato Etico della Romagna (protocol 5244/2019) and was carried out in accordance with the principles laid down in the 1964 Declaration of Helsinki. Primary leukemic cells were isolated using Lymphosep (Biowest, Nuaillé, France) from the bone marrow of adult newly diagnosed B-ALL patients (*n* = 3, Supplementary Table 1) and were seeded in RPMI-1640 Advance (Thermo Fisher Scientific, Waltham, MA, USA) supplemented with 20% FBS (GE Healthcare, Piscataway, NJ, USA).

### Compounds and drug schedule

Prexasertib (PX) and VE-821 were purchased from Medchemexpress and were dissolved in dimethyl sulfoxide (DMSO, Sigma-Aldrich, St. Louis, MO, USA) as 10-mM stocks and stored at −20 °C. Dox was kindly provided by the Oncology Pharmacy Unit, IRCCS Istituto Romagnolo per lo Studio dei Tumori “Dino Amadori”, Meldola, Italy. Dox stock solution (2 mg/ml) was stored at 4 °C and diluted directly in culture medium. Dox effect in single agent was evaluated seeding ALL cell lines at 0.5 × 10^6^ cell/ml and treating them with increasing drug concentrations (RPMI-8402 from 5 to 0.25 μM, dilution 1:2; SUP-B15 and REH from 1 to 0.05 μM, dilution 1:2). In the combination studies, ALL cell lines were seeded at 0.5 × 10^6^ cells/ml and were treated with Dox (RPMI-8402: 0.1 μM; SUP-B15 and REH: 0.05 μM) for 48 h. After that, cells were reseeded at 0.5 × 10^6^ cells/ml and treated with different doses of PX or VE-821 (or DMSO, as negative control), for additional 3 or 24 h depending on the experimental design.

### Cell viability and combination index analysis

Cell viability of ALL cell lines was quantified using CellTiter 96® AQueous One Solution Cell Proliferation Assay (Promega, Madison, WI, USA), following the manufacturer’s instructions. Absorbance was measured at 440 nm using a Labsystem Multiskan EX (Thermo Fisher Scientific). Cell viability of primary leukemic ALL cells was analyzed using the RealTime-Glo™ MT Cell Viability Assay (Promega) following the manufacturer’s recommendations. Luminescence was measured using a Glomax microplate luminometer (Promega). Viability reduction was expressed as a percentage of the controls (normalized to 100%). The additive, synergistic, and antagonistic effect of the drug combinations was evaluated according to the Chou-Talalay equation (Chou 2010), using Compusyn Software (ComboSyn Incorporated, Paramus, NJ, USA). Based on developer instructions, we defined the following: synergism where CI < 1; additivity where CI = 1; and antagonism where CI > 1.

### Cell cycle analysis by flow cytometry

Cell lines were harvested, fixed with 70% ice-cold ethanol, and stained using the PI staining mix (BD Biosciences, Franklin Lakes, NJ, USA). Flow cytometric analysis was performed on a BD FACSCanto II instrument (BD Biosciences) and cell cycle profile analysis was performed using ModfiT software (Verity Software House, ME, USA).

### Apoptosis detection by flow cytometry

Cell lines were stained by Annexin V/propidium iodide (PI) (eBioscience™ Annexin V Apoptosis Detection Kit FITC, Thermo Fisher Scientific) according to the manufacturer’s instructions. The percentage of Annexin V^+^ cells was determined on a FACS analyzer BD FACSCanto II (BD Biosciences) by assaying a minimum of 10,000 cells.

### Cell growth assay

To assess the effect of the combination on proliferation ability, cells were seeded at a concentration of 0.5 × 10^6^ cells/ml and treated with Dox (RPMI-8402: 0.05 μM; SUP-B15 and REH: 0.025 μM) for 48 h (day 0). Cells were then harvested, reseeded at 0.2 × 10^6^ cells/ml in fresh culture medium with subtoxic concentration of PX or VE-821 or DMSO, and counted using trypan blue exclusion assay (Sigma-Aldrich) every 72 h for 9 days of continuous drug exposure (D + 3, D + 6, and D + 9).

### Immunofluorescence analyses of mitotic alterations and chromosomes integrity

To assess the effect of the combination on mitotic alterations, ALL cell lines were treated with Dox for 48 h and then with PX or VE-821 (or DMSO) for further 3 h. Cells were seeded on poly-d-lysine-coated coverslips, fixed with 4% PFA. Blocking was performed with 1% BSA and 0.3% Triton X-100 in 1X PBS for 1 h. Slides were incubated overnight at 4 °C with anti-pericentrin (mouse monoclonal, 1:1000, ab28144 Abcam) and anti-γ-tubulin (rabbit polyclonal, 1:200, ab16504 Abcam) antibodies, washed, and stained respectively with goat anti-mouse Alexa Fluor 594 and goat anti-rabbit Alexa Fluor 488 secondary antibodies (1:500, Invitrogen) for 1 h at room temperature. The samples were washed three times in 1X PBS and mounted using ProLong Antifade DAPI (Invitrogen). Cells were imaged with a N-SIM E laser confocal microscope (Nikon Corporation) at a magnification of 60× and analyzed with NIS Elements software 5.11 (Nikon Corporation).

To assess the effect of the combination on chromosome integrity (Howe et al. 2014), cell pellets were resuspended in 0.075 M KCl, incubated at 37 °C for 15 min, centrifuged, and fixed with 10 ml of Carnoy’s Fixative (methanol:acetic acid, 3:1) three times. After the third centrifuge, the resuspension was dropped on chilled glass slides and chromosomes spreads were stained with ProLong Antifade DAPI (Invitrogen) following manufacturer’s instruction. The slides were analyzed using EVOS Fluorescence Microscope (AMG, Bothell, WA, USA) at a magnification of 100 × .

To quantify mitotic index, slides were analyzed using EVOS Fluorescence Microscope (AMG, Bothell, WA, USA) at a magnification of 40×. All quantitative analysis were performed using ImageJ software 1.52a (National Institutes of Health, NIH, USA). Mitotic cells were considered as cells presenting metaphase plates. Five different fields with an average number of 200–250 cells were examined in all cases and the mitotic index was calculated using the formula:

$$\mathrm{Mitotic}\ \mathrm{index}=\frac{\left(\mathrm{average}\ \mathrm{number}\ \mathrm{of}\ \mathrm{metaphases}\right)}{\left(\mathrm{average}\ \mathrm{total}\ \mathrm{number}\ \mathrm{of}\ \mathrm{cells}\right)}\times 100$$

### qRT-PCR

RNA was extracted from cells lysed in TRIzol® (Invitrogen) and was reverse transcribed into cDNA (PrimeScript Reag Kit with gDNA Eraser, Takara). qRT-PCR was performed on the 7500 Real Time PCR System (Applied Biosystems, ThermoFisher Scientific) by TaqMan gene expression assays (ThermoFisher Scientific) for CDK1 (Hs00938777_m1), CCNβ1 (Hs01030099_m1), CHEK1 (Hs00967506_m1), ATR (Hs00992123_m1), CHEK2 (Hs00200485_m1), and ATM (Hs00175892_m1). HPRT1 (Hs02800695_m1) was used as reference gene. Gene expression was quantified by the 2^−∆∆Ct^ method. The results are presented as ratio between normalized expression of the gene of interest in the target and in the DMSO-treated samples.

### Cell lysis and immunoblotting

Whole cell lysates were prepared in RIPA lysis buffer (Santa Cruz Biotechnology, TX, USA). Protein extracts were resolved by SDS-PAGE using Mini-Protean TGX stain-free precast gels and then electron-transferred onto nitrocellulose membranes (Bio-Rad Trans-blot turbo transfer pack). The following antibodies were used: anti-ATM (#2873), anti-pATM (Ser1981, #13050), anti-ATR (#2790), anti-pATR (Thr1989, #30632), anti-Chk1 (#2345), anti-pChk1 (Ser317, #2344), anti-Chk2 (#2662), anti-pChk2 (Thr68, #2197), anti-Cdc2 (#9112), anti-pCdc2 (Tyr15, #4539), and anti-CCNB1 (#4138), all from Cell Signaling Technology (Cell Signaling Technology, MA, USA) and anti-β-actin (ID, Sigma, St. Louis, MO). Horseradish peroxidase-conjugated anti-rabbit (NA934) IgG (GE Healthcare) was used as secondary antibody. The enhanced chemiluminescence kit SuperSignal™ West Femto (ThermoFisher Scientific) was used for signal detection at ChemiDoc-It (UVP). Data were analyzed by ImageJ 1.52v software (NIH).

### Statistical analysis

Data were presented as the mean ± standard deviation (SD) from at least three independent experiments performed in triplicates. Comparisons between two groups were performed using the Student’s *t* test, whereas multiple comparisons were performed using two-way analysis of variance with Dunnett post hoc test. *p* value <0.05 was considered as statistically significant difference. Statistical analysis was performed with Graphpad5 software (GraphPad Inc.).

## Results

### Subtoxic concentrations of Dox activate the G2/M cell cycle checkpoint in ALL cell lines

**Fig. 1:**
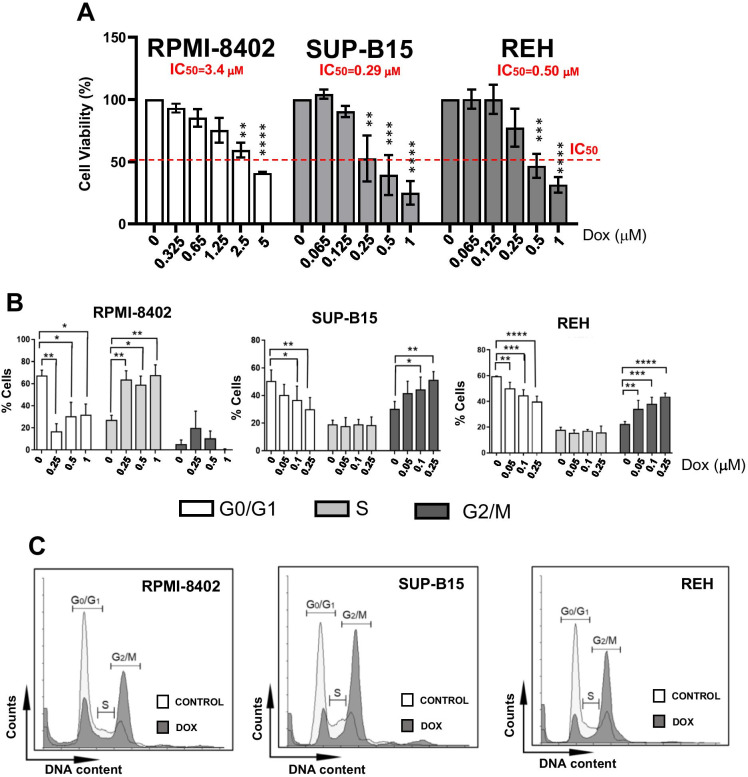
Subtoxic concentrations of Dox activate the G2/M cell cycle checkpoints in ALL cell lines. **A** Histograms showing cell viability analysis of RPMI-8402, SUP-B15, and REH cells treated with increasing concentration of Dox (RPMI-8402 from 5 to 0.25 μM, dilution 1:2; SUP-B15 and REH from 1 to 0.05 μM, dilution 1:2) for 24 h (*p < 0.05; **p < 0.01; ***p < 0.001). **B** Cell cycle analyses of RPMI-8402, SUP-B15, and REH cells treated with increasing concentration of Dox for 24 h. In the graph, the bars represent the mean ± standard deviation of at least three independent experiments (*p ≤ 0.05; **p ≤ 0.01; ***p ≤ 0.001). **C** Representative cell cycle graphs of RPMI-8402, SUP-B15, and REH treated with or without subtoxic concentrations of Dox (RPMI-8402, 0.1 μM; SUP-B15 and REH, 0.05 μM) for 48 h. Cell cycle profile of control and Dox-treated cells are represented in white and gray, respectively

To evaluate the effect of Dox on cell viability, we treated three B−/T-ALL cell lines with increasing concentrations of the compound for 24 h. Dox reduced the cell viability in a dose-dependent manner with RPMI-8402 cells being the least sensitive (IC_50_ = 3.4 μM) and SUP-B15 the most sensitive ones (IC_50_ = 0.29 μM, Fig. 1A). To investigate whether Dox affects cell cycle progression, cells were treated for 24 h with increasing concentrations of Dox based on the observed sensitivity. The treatment caused a progressive reduction of cells in the G0/G1 cell cycle phase and an increase in the percentage of cells in S (RPMI-8402 cells) or G2/M phase (REH and SUP-B15 cells) in a dose-dependent manner (Fig. 1B). Based on these results, we hypothesized that the activation of the cell cycle checkpoints could be a key survival mechanism of the leukemic cells and, consequently, the inhibition of the kinases involved in the regulation of S and G2/M phase checkpoint could increase Dox efficacy. Therefore, we sought to identify the lowest dose and time of exposure able to activate the cell cycle checkpoint without severe induction of cell apoptosis. We observed a significant G2/M checkpoint activation by treating ALL cell lines with subtoxic concentrations of Dox (0.1 μM for RPMI-8402; 0.05 μM for REH and SUP-B15 cells) for 48 h (Fig. 1C).

### The pharmacological inhibition of ATR kinase enhances the cytotoxicity of Dox in ALL cell lines

**Fig. 2:**
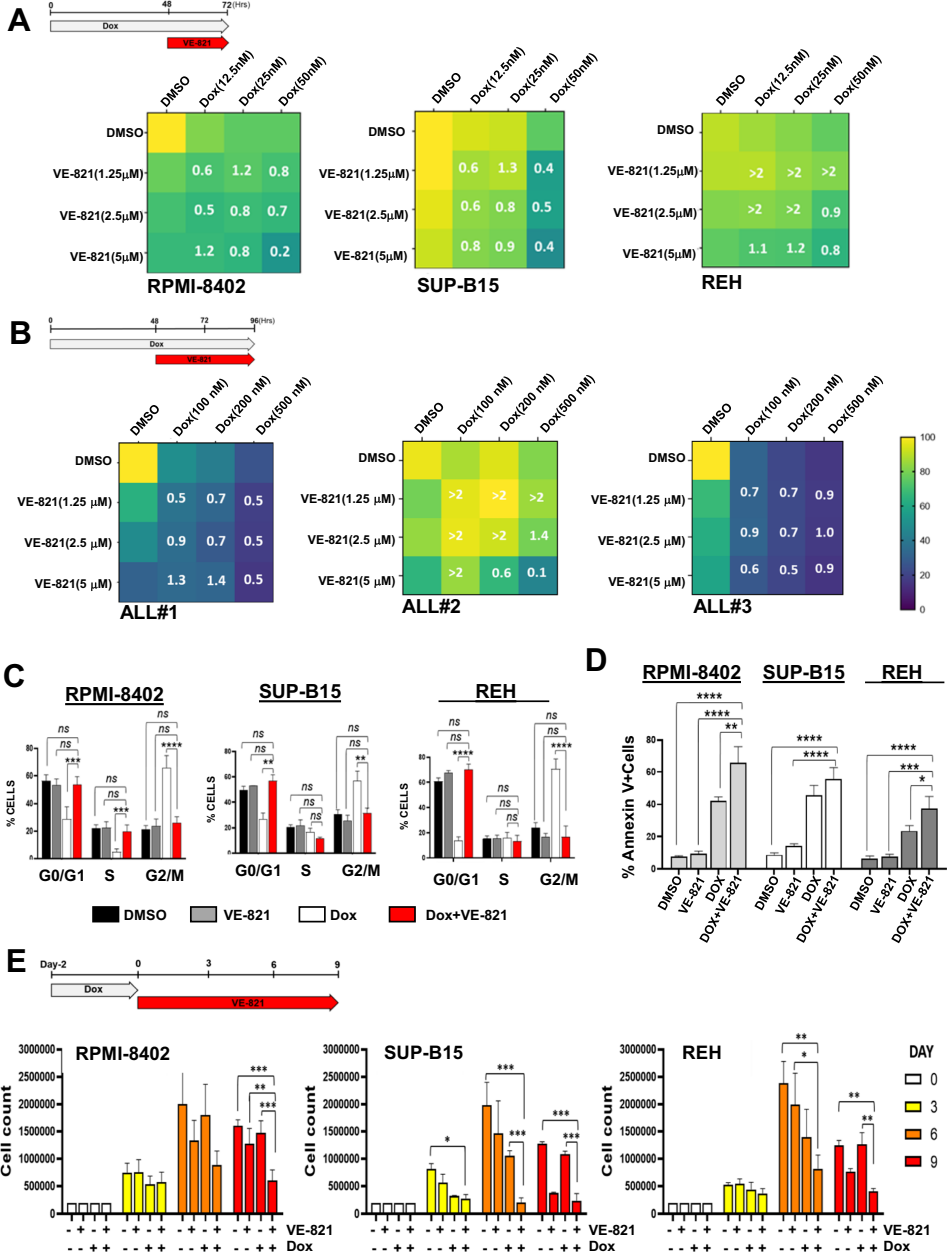
The pharmacological inhibition of ATR kinase enhances Dox cytotoxicity in ALL cells. **A** Heatmaps showing the effect of subtoxic concentrations of Dox for 48 h in combination with VE-821 for additional 24 h on cell viability of RPMI-8402, SUP-B15, and REH cells. **B** Heatmaps showing the effect of subtoxic concentrations of Dox for 48 h in combination with VE-821 for additional 48 h of primary ALL leukemic cells (*n* = 3). In the heatmaps, colors scale represents the values of mean normalized cell viability (% of cell viability relative to control) and numbers are combination index (CI) values. **C** Histograms showing the percentage of cells across cell cycle phases and **D** the percentage of Annexin V^+^ cells after treatment with subtoxic concentration of Dox (RPMI-8402, 0.1 μM; SUP-B15 and REH, 0.05 μM) for 48 h and then with VE-821 (5 μM) for additional 24 h. **E** Histograms representing the absolute number of cells during treatment with Dox (RPMI-8402, 0.1 μM; SUP-B15 and REH, 0.05 μM) for 48 h and then with or without VE-821 (5 μM) for further 9 days. Bars in **C**–**E** represent the mean ± standard deviation of at least three independent experiments (*p < 0.05; **p < 0.01; ***p < 0.001). The drug schedule is reported in the top left of **A**, **B**, and **E**

Double-strand breaks caused by Dox (Yang et al. 2014) is at the base of its synergic activity in combination with ATM inhibitors (Kurz et al. 2004; Zhu et al. 2014). However, this is not the only Dox mechanism of action. Indeed, inhibition of topoisomerase II enzymes by Dox, which promotes replicative stress (Yang et al. 2014), contributes to its cytotoxic activity. Therefore, we evaluated the consequences of ALL cells consecutive exposure to Dox and the selective ATR inhibitor VE-821, which inhibits the response to DNA SSBs. The inhibition of ATR kinase significantly enhanced Dox cytotoxicity in ALL cell lines (Fig. S1A). Combination index analyses confirmed a synergic (CI = 0.2) or additive (CI = 1.3) effect of the drug combinations in RPMI-8402 and SUP-B15 cells (Fig. 2A). REH cells showed a drug synergism only at the highest doses, while an antagonistic effect was observed at low doses (Fig. 2A). We tested the treatment schedule on primary leukemic cells from three ALL patients. We confirmed that the addition of VE-821 enhances Dox cytotoxicity in primary leukemic cells and induces strong synergism or additivity in terms of cell viability reduction, despite some heterogeneity among the three primary cases (Fig. 2B, S1B, and S1C). To understand the mechanism of action of the drug combination, we performed cell cycle analysis. Exposure of Dox-pretreated cells (G2/M arrested) to VE-821 restored the cell cycle profile to a distribution resembling control cells. Indeed, the number of cells arrested in the G2/M phase was significantly reduced after the addition of VE-821 in all the cellular models (Fig. 2C). We then performed apoptosis analysis to evaluate the cytotoxic effect of the combination. Treatment of Dox-exposed cells with VE-821 enhanced apoptosis in RPMI-8402 and REH cells (Fig. 2D). No significant differences were observed in SUP-B15 cells between treatment with Dox only and its combination with VE-821. Finally, to evaluate the effect of the combination during time, cells were incubated with Dox for 48 h and then with a subtoxic dose of VE-821 (2.5 μM) for further 9 days. The combination induced a reduction in the number of viable cells that became significant at 6 and/or 9 days of culture (Fig. 2E). In this experimental setting, SUP-B15 cells were the most sensitive cells, in which the combination completely abrogated cell growth (Fig. 2E).

### The inhibition of CHK1 enhances the biological effect of Dox in ALL cell lines

**Fig. 3:**
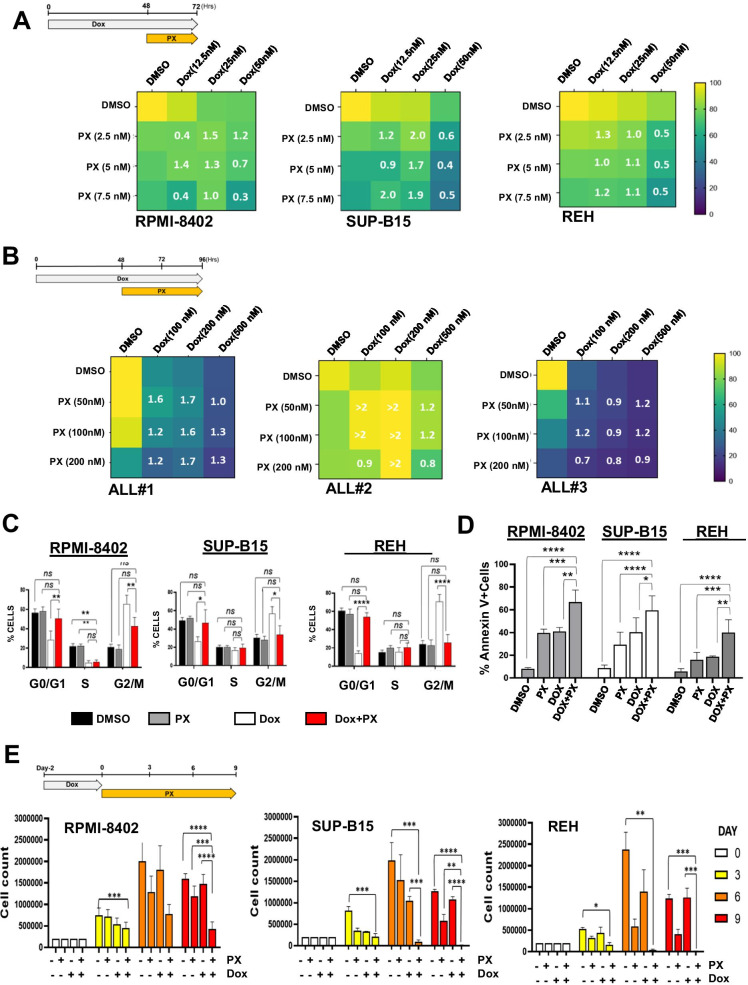
The pharmacological inhibition of CHK1 kinase enhances Dox cytotoxicity in ALL cells. **A** Heatmaps showing the effect of subtoxic concentrations of Dox for 48 h in combination with VE-821 for additional 24 h on cell viability of RPMI-8402, SUP-B15, and REH cells. **B** Heatmaps showing the effect of subtoxic concentrations of Dox for 48 h in combination with VE-821 for additional 48 h of three primary ALL leukemic cells. In the heatmaps, colors scale represents the values of mean normalized cell viability (% of cell viability relative to control) and numbers are combination index values. **C** Histograms showing the percentage of cells across cell cycle phases and **D** the percentage of Annexin V^+^ cells after treatment with subtoxic concentration of Dox (RPMI-8402, 0.1 μM; SUP-B15 and REH, 0.05 μM) for 48 h and then with PX (RPMI-8402: 0.0075 μM; SUP-B15: 0.03 μM; REH:0.05 μM) for additional 24 h. **E** Histograms representing the absolute number of cells during treatment with Dox (RPMI-8402, 0.1 μM; SUP-B15 and REH, 0.05 μM) for 48 h and then with or without PX (RPMI-8402: 0.0075 μM; SUP-B15: 0.03 μM; REH: 0.05 μM) for further 9 days. Bars in **C**–**E** represent the mean ± standard deviation of at least three independent experiments (*p < 0.05; **p < 0.01; ***p < 0.001). The drug schedule is reported in the top left of **A**, **B**, and **E**

In response to replicative stress, ATR phosphorylates and activates CHK1 kinase which, in turn, promotes cell cycle arrest. However, during the DDR, CHK1 is also activated by ATM kinase (Moiseeva et al. 2019). Thus, we investigated the effect of the combination between Dox and CHK1 functional inhibition, by using a selective CHK1 inhibitor (prexasertib, PX) able to inhibit the activation of CHK1 downstream targets (di Rorà et al. 2016). As showed by the viability and by the combination index analyses, PX enhanced Dox cytotoxicity in ALL cell lines, in a synergic or additive way (Fig. 3A and S2A). An additive and/or weak synergic effect of PX and Dox was also observed in primary ALL cells, in term of reduction of the cell viability, despite some heterogeneity among samples (Fig. 3B, S2B, and S2C). In line with the results obtained by the combination of Dox and VE-821, Dox treatment followed by PX exposure restored the cell cycle profile to a distribution resembling control cells (by significantly reducing the percentage of G2/M cells, Fig. 3C) and significantly increased the number of apoptotic cells in comparison with single agent treatments (Fig. 3D). We then tested the combination between Dox and PX in terms of reduction of proliferative capacity overtime. The combination dramatically reduced (in RPMI-8402) or completely abrogated (SUP-B15 and REH) cell growth in comparison with single agent treatments (Fig. 3E).

### The inhibition of the ATR-CHK1 pathway compromises mitotic regulation in Dox-pretreated ALL cell lines and induces aberrant chromosome segregation and mitotic spindle defects

**Fig. 4:**
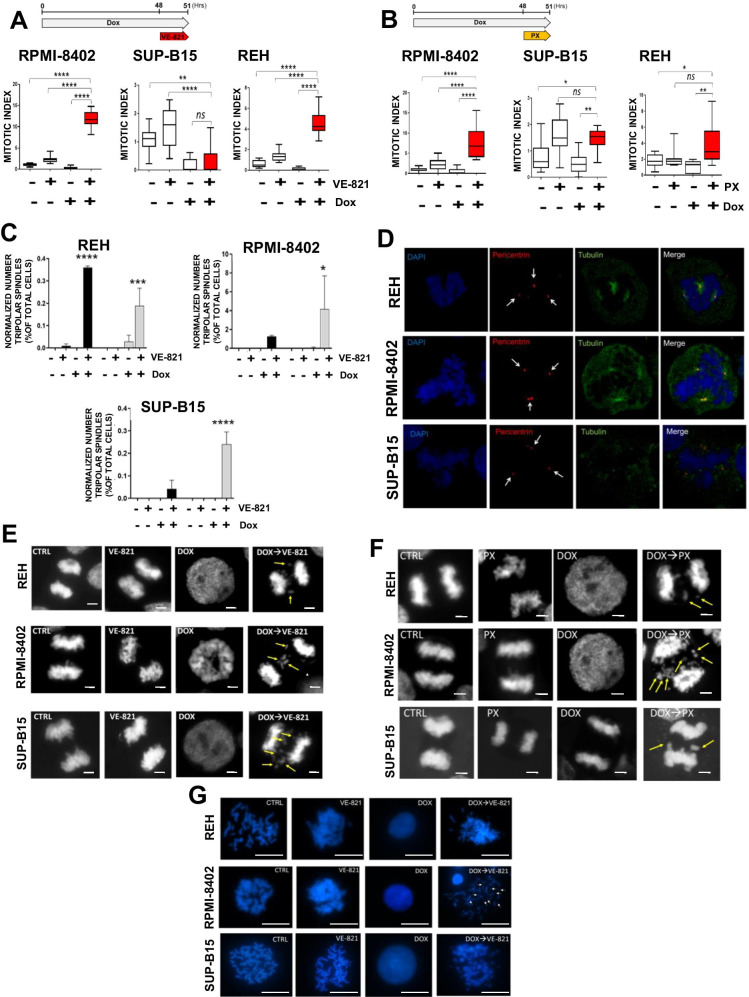
The inhibition of the ATR-CHK1 pathway compromises mitotic regulation in ALL cell lines. **A** Box and whiskers plots representing mitotic index analysis of RPMI-8402, SUP-B15, and REH cells treated with Dox (RPMI-8402; 0.1 μM; SUP-B15 and REH: 0.05 μM) for 48 h and then with VE-821 (5 μM) or **B** PX (RPMI-8402: 0.0075 μM; SUP-B15: 0.03 μM; REH: 0.05 μM) for additional 3 h. Mitotic indices have been calculated as follows: (average number of mitotic/average total number of cells) * 100. **C** Histograms representing the normalized number of tripolar spindles expressed as percentage of total cell count. In the graph, black and gray bars represent Dox + VE-821 and Dox + PX combinations, respectively. **D** Immunofluorescence analysis showing tripolar spindles in RPMI-8402, SUP-B15, and REH cells treated with Dox (RPMI-8402: 0.1 μM; SUP-B15 and REH: 0.05 μM) for 48 h and then with VE-821 (5 μM) for additional 3 h. In the picture, cells were stained with DAPI (blue), anti-tubulin antibody (green), and anti-pericentrin antibody (red). Scale bar: 5 μm. **E** DAPI labeled DNA showing lagging chromosomes in RPMI-8402, SUP-B15, and REH cells treated with Dox (RPMI-8402: 0.1 μM; SUP-B15 and REH: 0.05 μM) for 48 h and then with VE-821 (5 μM) or **F** PX (RPMI-8402: 0.0075 μM; SUP-B15: 0.03 μM; REH: 0.05 μM) for additional 3 h. In the pictures, lagging chromosomes are pointed by yellow arrows. Scale bar: 5 μm. **G** DAPI labeled DNA showing chromosomes integrity analysis of RPMI-8402, REH, and SUP-B15 cells treated with Dox and then with VE-821. In the figures, fragmented chromosomes are pointed by white arrows. Scale bar: 20 μm. In the figures, statistical significance was represented as asterisks (*p < 0.05; **p < 0.01; ***p < 0.001)

To understand the fate of ALL cell lines following G2/M cell cycle checkpoint override, we performed immunofluorescence analysis on Dox-arrested cells treated for 3 h with VE-821 or PX. The combination of Dox and VE-821 significantly enhanced the mitotic index in RPMI-8402 and REH cells, but not in SUP-B15 cells (Fig. 4A and S3A). A similar increase of mitotic index was observed in the three models by combining Dox and PX (Fig. 4B and S4A). Several mitotic defects were found in the cell lines under the pressure of one or the other drug combination, including DNA bridges, metaphases with lagging chromosomes, and a significant number of tripolar spindles. Both combinations were able to induce tripolar spindles, but a higher number was found in cells treated with Dox and PX (Fig. 4C, S3A, and S4A). By co-staining with anti-γ-tubulin and anti-pericentrin antibodies, we showed that the tripolar spindles generated by the combinations were characterized an abnormal number of kinetochores (*n* > 2, Fig. 4D).

The inhibition of ATR or CHK1 kinase in Dox-pretreated cells compromised the correct chromosomes segregation. Indeed, several metaphases with lagging chromosomes or chromatin bridges were observed in the cells treated with Dox and VE-821 (Fig. 4E and S3A) or Dox and PX (Fig. 4F and S4A). Finally, we evaluated whether chromosome mis-segregation during metaphases was associated with their structural aberrations. Chromosome integrity analysis of cells treated with Dox and VE-821 revealed the presence of fragmented chromosomes in the combination treatment, which were not detected in single agent treatments and in control cells (Fig. 4G).

### The inhibition of the ATR-CHK1 pathway induces G2/M checkpoint override in Dox-arrested ALL cell lines

**Fig. 5:**
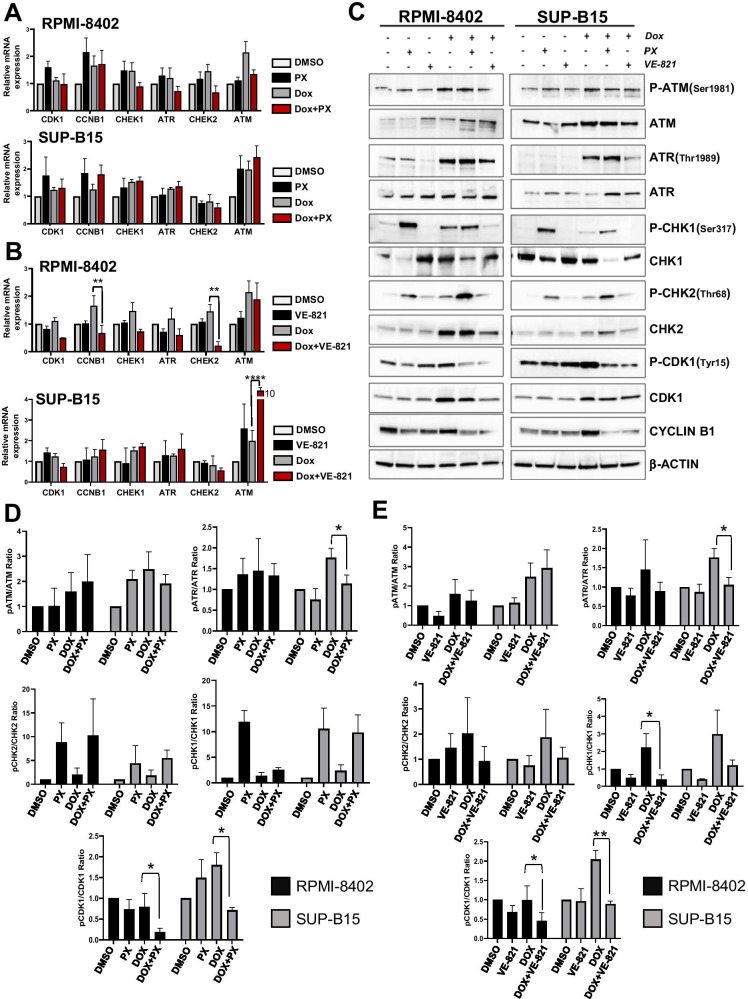
The pharmacological inhibition of the ATR-CHK1 kinases causes G2/M checkpoint override in ALL cell lines. **A** Relative mRNA expression of *CCNB1*, *CDK1*, *CHK1*, *CHK2*, *ATM*, and *ATR* genes in RPMI-8402 and SUP-B15 cells treated with Dox (0.1 and 0.05 μM, respectively) for 48 h and with PX (0.0075 and 0.03 μM, respectively) or **B** VE-821 (5 μM) for further 3 h. In the graph, bars represent the mRNA level normalized on the control sample. The mean ± standard deviation of three independent experiments is shown. **C** Representative western blots of RPMI-8402 and SUP-B15 cells treated with Dox for 48 h and with PX or VE-821 for further 3 h. β-Actin was used for loading normalization. **D** Histograms showing ratio of pATR/ATR, pATM/ATM, pCHK1/CHK1, pCHK2/CHK2, and pCDK1/CDK1 protein levels in RPMI-8402 (black) and SUP-B15 (gray) cells treated with Dox (0.1 and 0.05 μM, respectively) for 48 h and with PX (0.0075 and 0.03 μM, respectively) or **E** VE-821 (5 μM) for further 3 h. Protein expression ratio is reported as the mean ± standard deviation of at least three independent experiments. In the figures, statistical significance was indicated by asterisks (*p < 0.05; **p < 0.01; ***p < 0.001)

To understand the molecular changes associated with the observed mitotic defects, we analyzed the expression of G2/M checkpoint-related genes in RPMI-8402 and SUP-B15 cells treated with Dox in combination with VE-821 or PX, using the same experimental conditions of immunofluorescence studies. The two cell lines were chosen based on the differential response to the combinations in term of changes in the mitotic index (Fig. 4A and B). We did not observe significant differences in the mRNA level of G2/M checkpoint-related genes between cells treated with Dox alone and in combination with PX (Fig. 5A). In parallel, we found a significant downregulation of *CCNB1* and *CHK2* transcripts in RPMI-8402 cells and a significant upregulation of *ATM* in SUP-B15 cells treated with Dox in combination with VE-821 (Fig. 5B). We then analyzed protein expression and activation levels. The two combinations significantly reduced the levels of pCDK1^Tyr15^ in both the cell lines (Fig. 5C, D, E, S5A, S5B, S5D, and S5E). Similar effect was seen in term of protein expression of Cyclin B1, key component of the CDK1-CyclinB1 complex (mitotic promoting factors (Ghelli Luserna Di Rorà et al. 2019)) (Fig. 5C and S5C). The reduction of Cyclin B1 expression together with the reduction of pCDK1^Tyr15^ confirmed that treatment with the two combinations can override the G2/M checkpoint. Regarding the ATR-CHK1 pathway, we found restoration of physiological levels of the pATR/ATR ratio (SUP-B15 significant reduction, RPMI-8402 trend of reduction) and the pCHK1/CHK1 ratio (RPMI-8402 significant reduction, SUP-B15 trend of reduction) in the samples treated with Dox and VE-821 in comparison to single agent Dox treatment. The reduction of pATR Thr1989 in the combined treatment is in line with the inhibitory effect of VE-821 on ATR functionality which is essential for ATR phosphorylation itself (Nam et al. 2011). The same changes were observed in the pATR/ATR ratio (SUP-B15 significant reduction), but not in the pCHK1/CHK1 ratios in the combination between Dox and PX (Fig. 5D, C, E, S5A, S5B, S5D, and S5E). Regarding the ATM-CHK2 pathway, we did not observe significant changes in terms of protein activation. However, both cell lines showed a trend towards higher pCHK2/CHK2 when treated with Dox and PX compared with Dox alone, suggesting a potential activation of the ATM-CHK2 signaling. Different results were seen in the samples treated with Dox and VE-821 in which the pCHK2/CHK2 ratio was decreased by the combination in comparison to Dox in both cell lines (Fig. 5D, C, E, S5A, S5B, S5D, and S5E).

## Discussion

In this study, we show evidence of the crucial role of the ATR-CHK1 pathway in the response to Dox and we demonstrate that its inhibition enhances Dox cytotoxicity against ALL cells. DDR kinases, such as ATR and CHK1, are crucial tumor suppressors in eukaryotic cells as they maintain the integrity of the genome and suppress tumorigenesis (Reddy et al. 2010; Sarmento and Barata 2016; Smith et al. 2010). However, in cancer cells, these kinases may act as pseudo-oncogenes (Filipponi et al. 2019; Sarmento and Barata 2016). Indeed, their over-expression, and exacerbated activity, may protect the genome of malignant cells from DNA damaging-based therapy (e.g., radiotherapy and chemotherapy). Cancer cells activate different DDR pathways in order to survive and continue to proliferate. For example, it has been reported that different solid and hematologic tumor models respond to Dox-induced DSBs activating the G2/M checkpoint in order to slow down cell cycle progression, to promote DNA repair, and to survive (Shin et al. 2015; Wang et al. 2018; Zimmermann et al. 2012). Accordingly, our data show that ALL cell lines respond to Dox exposure by activating the G2/M cell cycle checkpoint. To override this mechanism and to enhance Dox efficacy, we designed different drug schedules that combine Dox with selective ATR-CHK1 pathway inhibitors.

The most effective combinations were obtained by exposure of Dox-pretreated cells to VE-821 or PX rather than by simultaneous treatment (data not shown). From a mechanistic point of view, the addition of VE-821 or PX in Dox-pretreated cells abrogated the G2/M cell cycle checkpoint and restored a cell cycle profile resembling untreated samples. Combination index and apoptosis analyses confirmed that the abrogation of cell cycle checkpoint was followed by a synergic reduction of cell viability that was confirmed in primary leukemic cells and by an additive induction of apoptosis. Of note, the drug schedules were able to interfere with leukemia cell proliferation for 9 days using a Dox washout after 48 h of treatment, followed by single drug exposure of PX or VE-821. This experimental setting, which caused a proliferation decrease in the control samples at day 9 likely due to medium exhaustion, was chosen in order to avoid potential confounding effect on cell proliferation due to cell medium replacement during the 9 days of culture.

Immunoblotting analysis performed on samples treated with Dox for 48 h and then with VE-821 or PX for further 3 h showed different perturbations of the ATR-CHK1 and ATM-CHK2 pathway depending of the cell lines. Dox treatment significantly increased the expression of Cyclin B1 and phosphor-CDK1 (tyr15) in RPMI-8402 and SUP-B15 cells, confirming the accumulation of cells in G2/M phase. The addition of VE-821 or PX significantly reduced the expression of both markers. Cyclin B1 expression is crucial to regulate mitotic entry/exit and physiologically its expression drops down during metaphase, when all kinetochores are attached to the fibers of the mitotic spindle (Ghelli Luserna Di Rorà et al. 2019). In this scenario, the inhibition of ATR or CHK1 kinases interfered with mitotic regulation by promoting mitotic exit even in the presence of arrest signals induced by Dox. The premature mitotic exit was confirmed by a significant increment of the mitotic index in ALL cell lines treated with the two combinations and, in particular, with Dox and PX. Moreover, the combinations generated cells with lagging chromosomes during cell division. Indeed, we detected a significant number of metaphases with lagging chromosomes and with chromatin bridges. These results are indicative of an opposite response compared to that induced by combining Dox with ATM or CHK2 inhibitors in cancer cell lines (Bakhoum et al. 2014). In normal epithelium cells (RPE1) and solid cancer cell lines, Dox induced metaphases with lagging chromosomes and the addiction of CHK2 or ATM inhibitors significantly reduced them to the level of untreated controls (Bakhoum et al. 2014). Moreover, our data show that both VE-821 and Dox alone were able to induce a small percentage of tripolar spindles, while the two combinations significantly increased the number of such mitotic alterations. The mechanism of tripolar spindles induction in the combined samples is still unknown.

## Conclusions

**Fig. 6:**
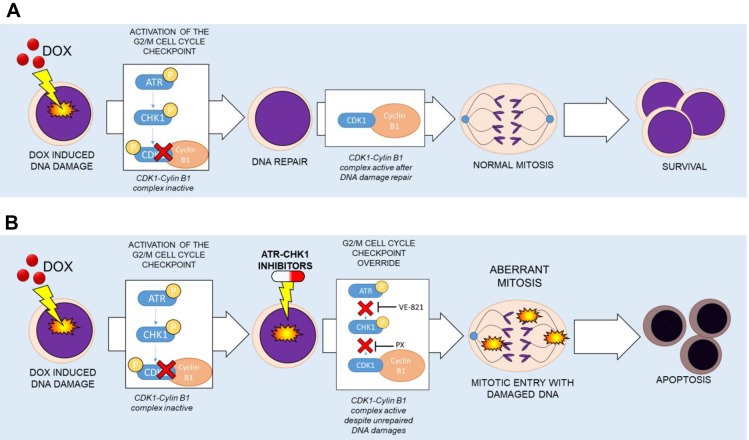
Schematic representation of the effect of ATR-CHK1 inhibitors in Dox damaged cells. **A** Graphical representation of the hypothetical mechanism of response to Dox in leukemic cells as monotherapy or **B** in combination with ATR-CHK1 inhibitors

Recently new formulations and novel combination strategies have been developed to enhance the clinical efficacy of Dox. These improved formulations, such as the liposomal one, increase Dox internalization in cancer cells while reducing the amount of compound needed to obtain therapeutic efficacy (Quarello et al. 2012). The main goal of these new therapeutic approaches is to reduce side effects (Hunault-Berger et al. 2011). Here, we demonstrated that our new in vitro drug schedule that combines Dox followed by ATR/CHK1 inhibitors can increase Dox cytotoxicity against ALL cells, while using lower drug doses (Fig. 6A and B).

### Supplementary Information

ESM 1 (DOCX 12 kb)

ESM 2 Fig. S1: Effect of Dox in combination with VE-821 on cell viability in ALL cell lines and primary leukemic ALL cells. **A)** Reduction of cell viability of RPMI-8402, SUP-B15 and REH cells treated with Dox for 48 h and with VE-821 for further 24 h. Bars represent the mean ± standard deviation of at least three independent experiments. Statistical significance of the comparison between each drug concentration and DMSO treated cells was indicated by asterisks (*p < 0.05; **p < 0.01; *p* < 0.001). **B)** Cell viability analysis of primary leukemic ALL cells (*n* = 3) treated with Dox for 48 h and then with increasing concentrations of VE-821 for further 24 h or **C)** 48 h. Fig. S2: Effect of Dox in combination with PX on cell viability in ALL cell lines and primary leukemic ALL cells. **A)** Reduction of cell viability of RPMI-8402, SUP-B15 and REH cells treated with Dox for 48 h and with PX for further 24 h. Bars represent the mean ± standard deviation of at least three independent experiments. Statistical significance of the comparison between each drug concentration and DMSO treated cells was indicated by asterisks (*p < 0.05; **p < 0.01; p < 0.001). **B)** Cell viability analysis of primary leukemic ALL cells (n = 3) treated with Dox for 48 h and then with increasing concentrations of PX for further 24 h or **C)** 48 h. Fig. S4: Induction of tripolar spindles in ALL cell lines treated with Dox and VE-821. **A**) Immunofluorescence analysis of RPMI-8402, SUP-B15 and REH cells treated Dox (RPMI-8402, 0.1 μM; SUP-B15 and REH, 0.05 μM) for 48 h and then with VE-821 (5 μM) for further 3 h. In the figures, metaphases are showed by white circles and tripolar spindles are showed by yellow triangles; scale bars indicate 200 μm. Fig. S4: Induction of tripolar spindles in ALL cell lines treated with Dox and PX. **A**) Immunofluorescence analysis of RPMI-8402, SUP-B15 and REH cells treated Dox (RPMI-8402, 0.1 μM; SUP-B15 and REH, 0.05 μM) for 48 h and then with PX (0.1, 0.25 and 0.5 μM) for further 3 h. In the figures, metaphases are showed by white circles and tripolar spindles are showed by yellow triangles; scale bars indicate 200 μm. Fig. S5: Effect of combination between Dox and VE-821 or PX in the ATR/CHK1 and ATM/CHK2 pathway **A-B**) Western blots of RPMI-8402 and SUP-B15 cells treated with Dox for 48 h and with PX or VE-821 for further 3 h. β-actin was used for loading normalization. **C**) Bands intensity analysis of RPMI-8402 and SUP-B15 cells treated with Dox for 48 h and then with VE-821 or PX for further 3 h. Bars represent the mean ± standard deviation of at least three independent experiments. Statistical significance was indicated only for Dox + PX or Dox + VE-821 versus Dox alone by asterisks (*p < 0.05; **p < 0.01). **D**) Bands intensity analysis of RPMI-8402 and SUP-B15 cells treated with Dox for 48 h and then with PX or **E)** VE-821 for further 3 h. Bars represent the mean ± standard deviation of at least three independent experiments. Statistical significance was indicated only for Dox + PX or Dox + VE-821 versus Dox alone by asterisks (*p < 0.05). (PDF 1219 kb)

## Data Availability

Not applicable.

## Abbreviations

ALL: Acute lymphoblastic leukemia
ATM: Ataxia-telangiectasia mutated
ATR: Ataxia- and Rad3-related
CDK1: Cyclin dependent kinase 1
CHK1: Checkpoint kinase 1
CHK2: Checkpoint kinase 2
CI: Combination index
DDR: DNA damage response
Dox: Doxorubicin
DSBs: Double-strand breaks
PX: Prexasertib
