# Correction to: Exploring the ATR-CHK1 pathway in the response of doxorubicin-induced DNA damages in acute lymphoblastic leukemia cells

**DOI:** 10.1007/s10565-024-09913-1

**Published:** 2024-08-31

**Authors:** Andrea Ghelli Luserna Di Rorà, Martina Ghetti, Lorenzo Ledda, Anna Ferrari, Matteo Bocconcelli, Antonella Padella, Roberta Napolitano, Maria Chiara Fontana, Chiara Liverani, Enrica Imbrogno, Maria Teresa Bochicchio, Matteo Paganelli, Valentina Robustelli, Seydou Sanogo, Claudio Cerchione, Monica Fumagalli, Michela Rondoni, Annalisa Imovilli, Gerardo Musuraca, Giovanni Martinelli, Giorgia Simonetti

**Affiliations:** 1 Biosciences Laboratory, IRCCS Istituto Romagnolo per lo Studio dei Tumori (IRST) “Dino Amadori”, Via Piero Maroncelli, 40, 47014 Meldola, FC Italy; 2 Department of Experimental, Diagnostic and Specialty Medicine, Institute of Hematology “L. e A. Seragnoli”, University of Bologna, Bologna, Italy; 3 Hematology Unit, IRCCS Istituto Romagnolo per lo Studio dei Tumori (IRST) “Dino Amadori”, Meldola, FC Italy; 4 Hematology Division and Bone Marrow Transplantation Unit, San Gerardo Hospital, Monza, Italy; 5 Hematology Unit, Ospedale Santa Maria delle Croci, Ravenna, Italy; 6 AUSL-IRCCS di Reggio Emilia, Reggio Emilia, Italy; 7 Scientific Directorate, IRCCS Istituto Romagnolo per lo Studio dei Tumori (IRST) “Dino Amadori”, Meldola, FC Italy

**Correction to** **:** **Cell Biol Toxicol (2023) 39:795–811**

10.1007/s10565-021-09640-x

The original version of this article unfortunately contained an error in the funding section.

Currently the text regarding the Funding is "The study was supported by Associazione Italiana per la Ricerca sul Cancro (AIRC, investigator grant ref. 19226) and by ERA-Per-Med (reference number: ERAPERMED2018-275)". The corrected statement is as follows. "The study was supported by Associazione Italiana per la Ricerca sul Cancro (AIRC, investigator grant ref. 23810) and by ERA-Per-Med (reference number: ERAPERMED2018-275)".

