# Supplementary material for: Exploring the ATR-CHK1 pathway in the response of doxorubicin-induced DNA damages in acute lymphoblastic leukemia cells
Source: Cell Biol Toxicol. 2021 Sep 14;39(3):795–811. doi: 10.1007/s10565-021-09640-x (PMC10406704; doi:10.1007/s10565-021-09640-x)
Supplement: Supplementary file 1 — (DOCX 12 kb) [file 10565_2021_9640_MOESM1_ESM.docx]

| SAMPLE ID | AGE | SEX | CYTOGENETIC CLASS |
| --- | --- | --- | --- |
| ALL#1 | 60 | female | Normal Karyotype |
| ALL#2 | 73 | male | Normal Karyotype |
| ALL#3 | 25 | male | t(1;19)(q23;p13) |

Table 1: Characteristics of primary B-ALL
