# Supplementary figures and images for: Exploring the ATR-CHK1 pathway in the response of doxorubicin-induced DNA damages in acute lymphoblastic leukemia cells
Source: Cell Biol Toxicol. 2021 Sep 14;39(3):795–811. doi: 10.1007/s10565-021-09640-x (PMC10406704; doi:10.1007/s10565-021-09640-x)

A.

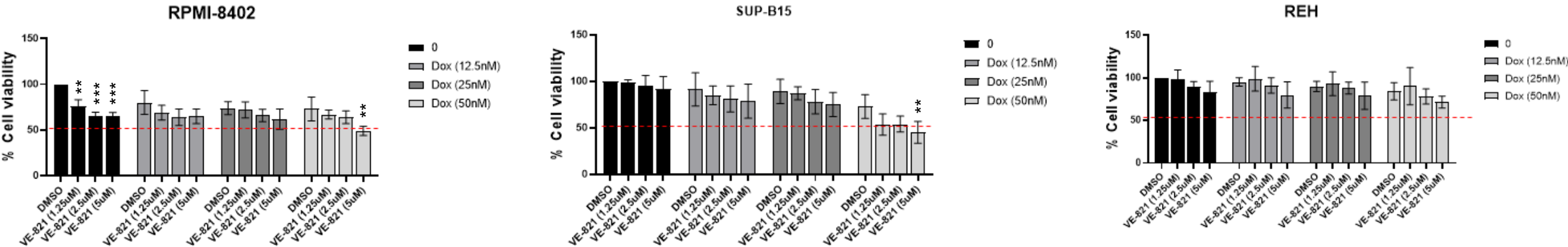

B.

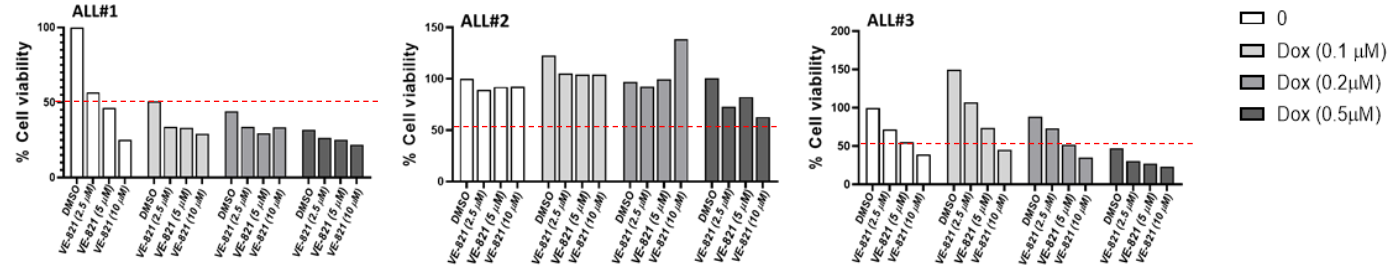

C.

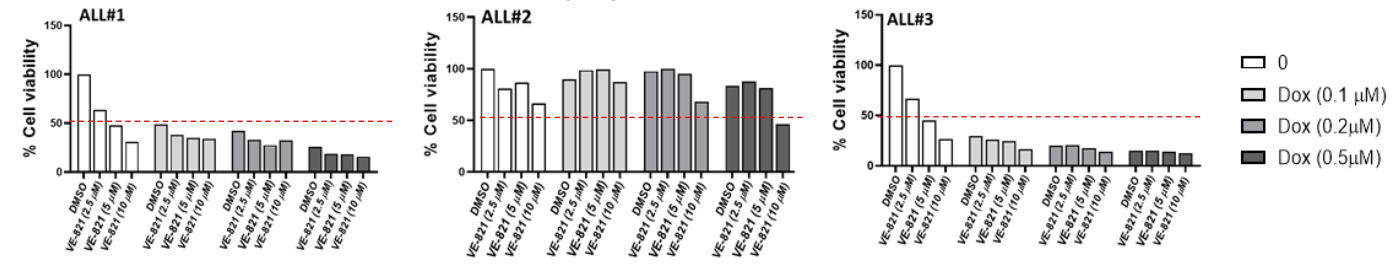

# A.

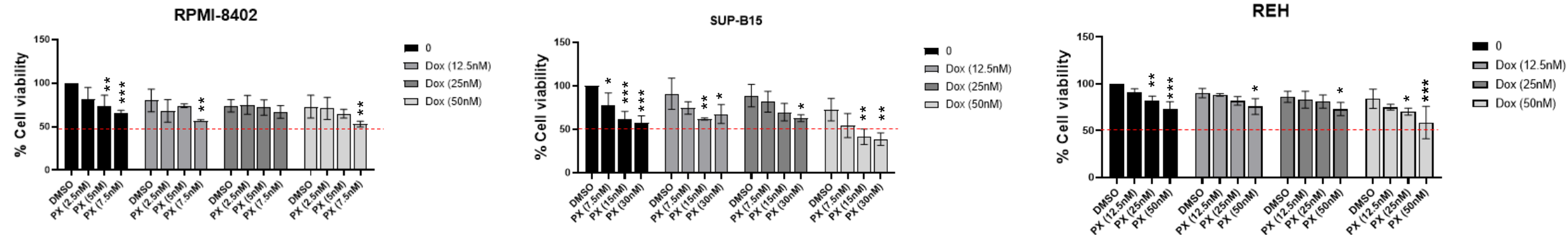

**B.**

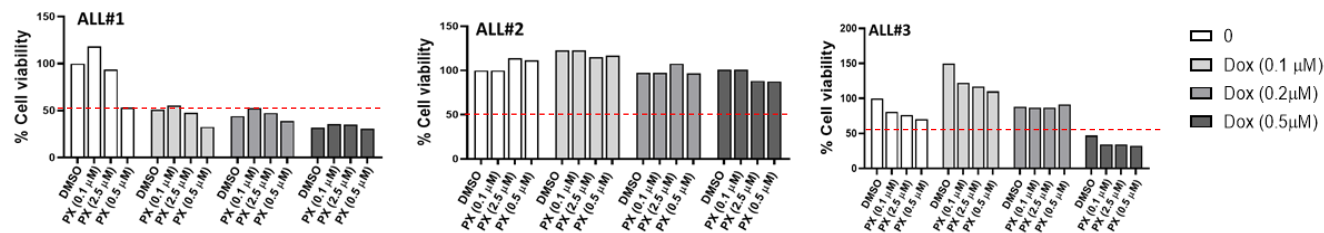

**C.**

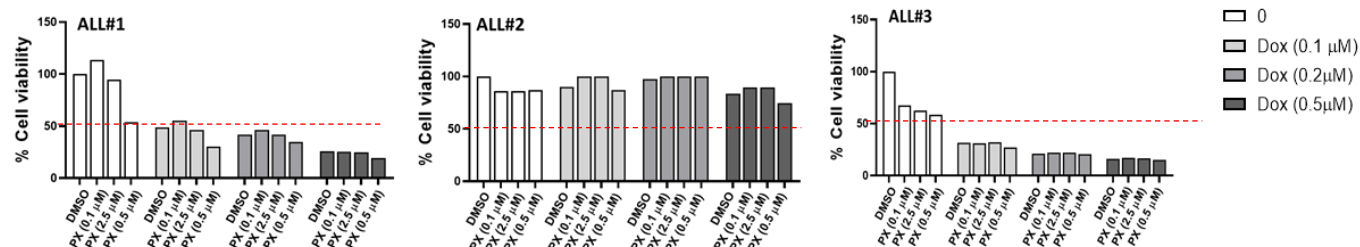

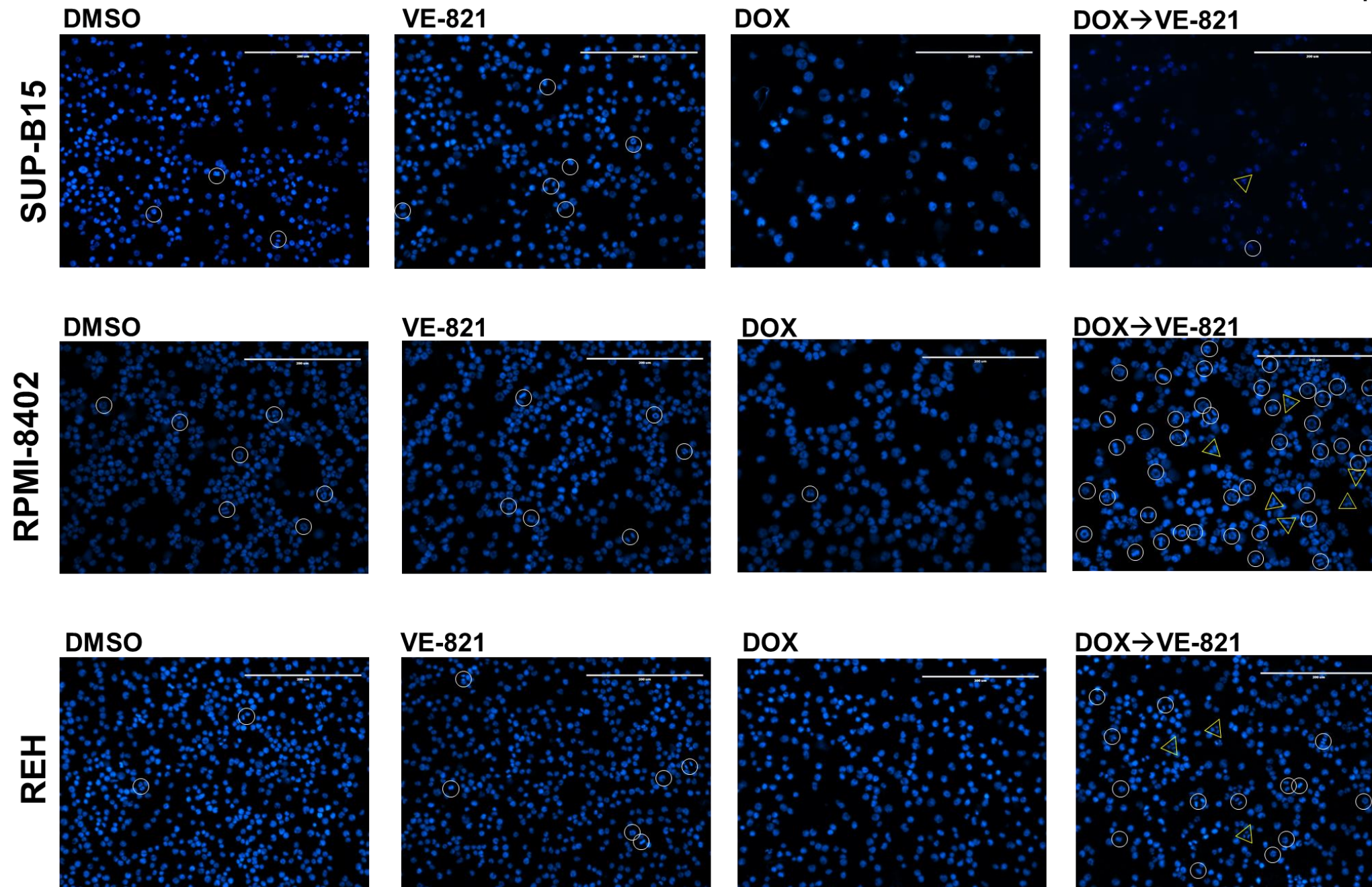

SUP-B15

DMSO

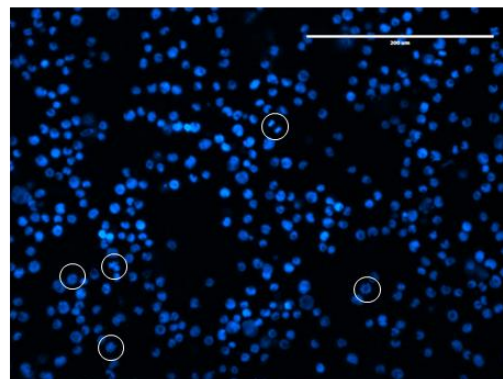

PX

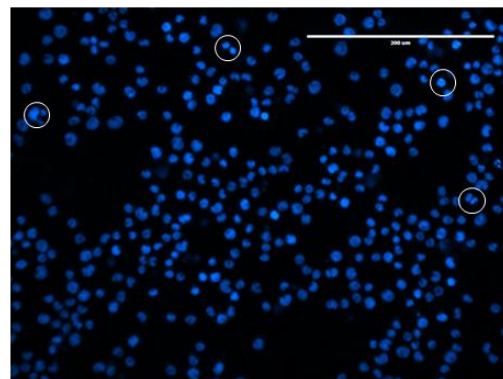

DOX

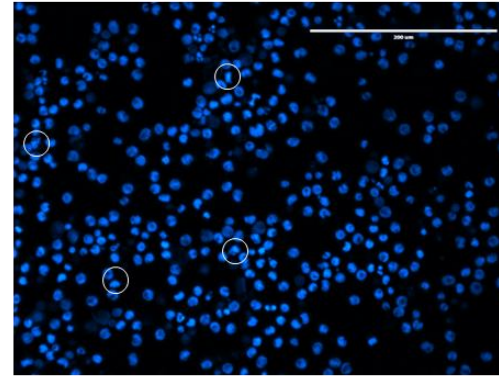

DOX→PX

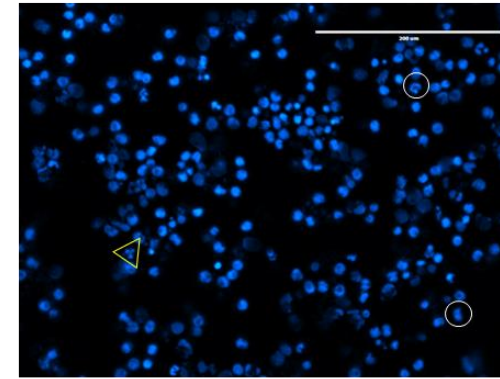

RPMI-8402

DMSO

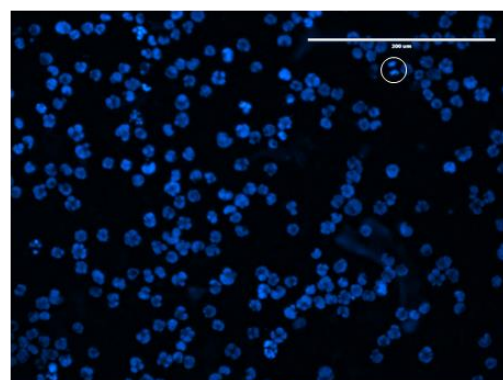

PX

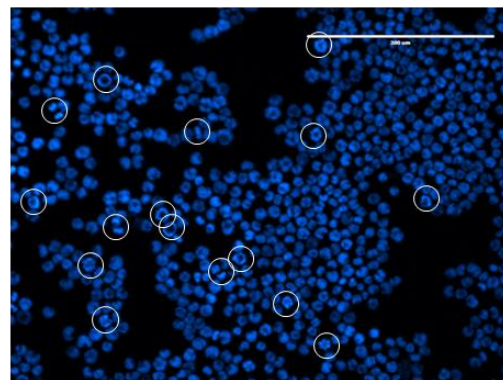

DOX

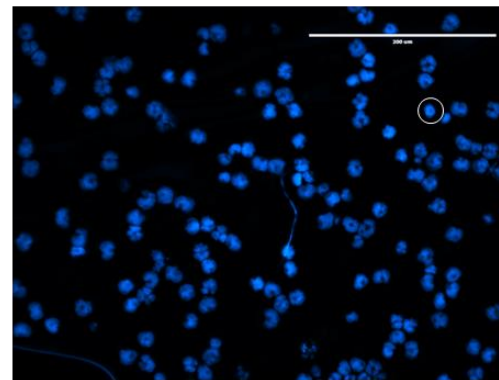

DOX→PX

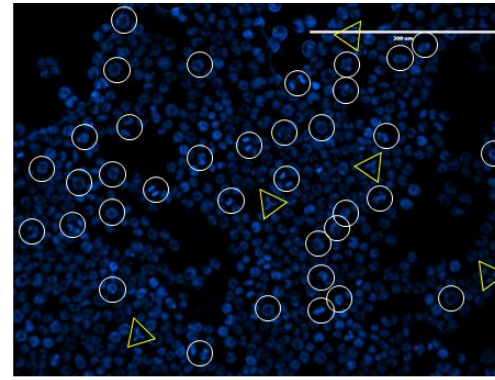

REH

DMSO

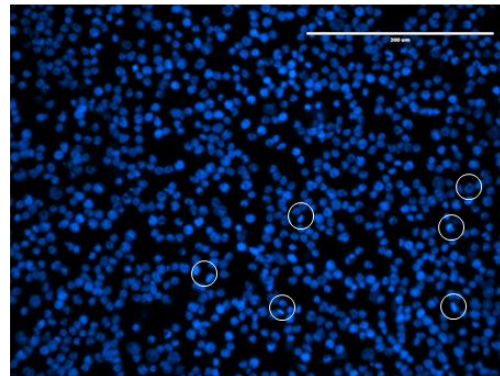

PX

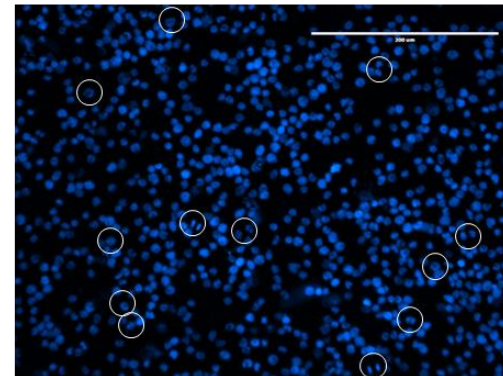

DOX

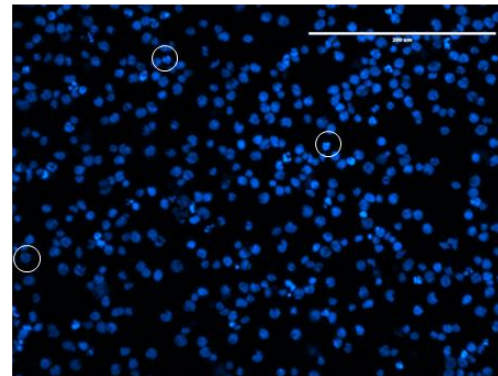

DOX→PX

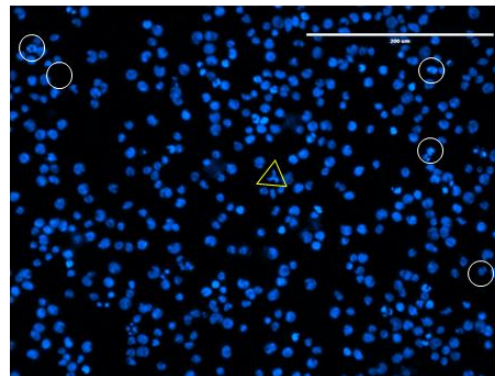

A.

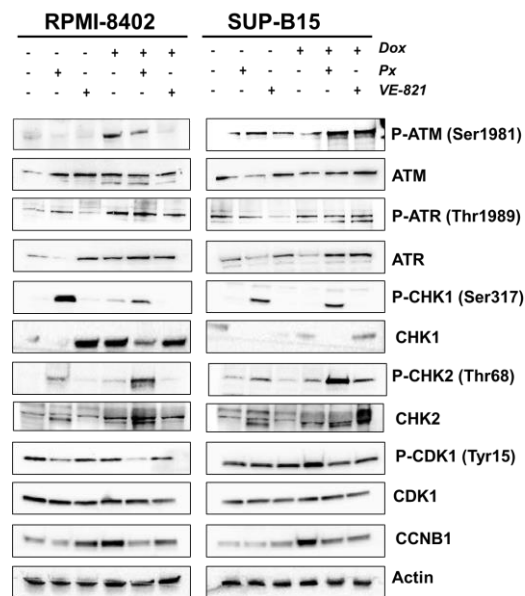

B.

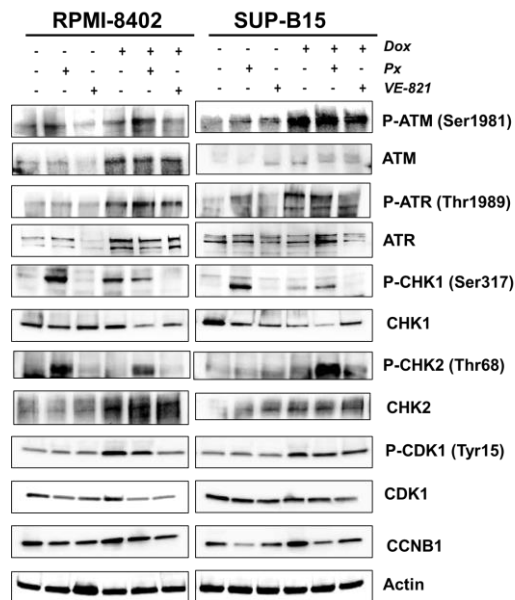

C.

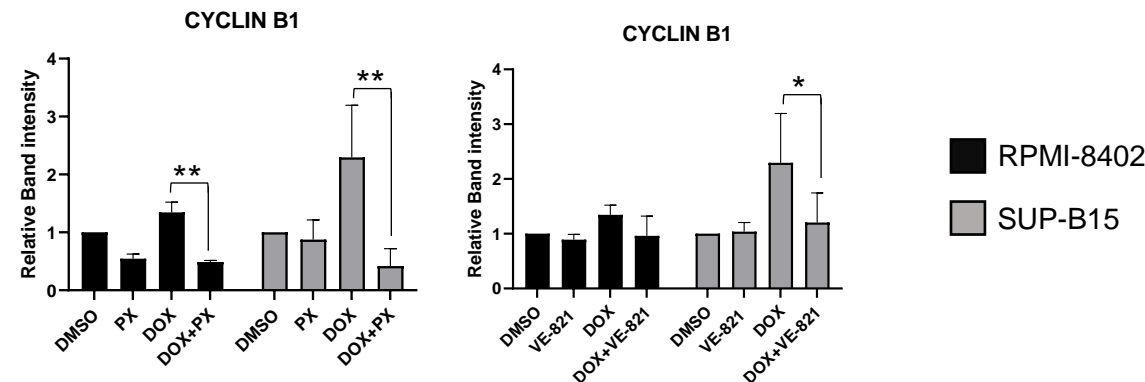

D.

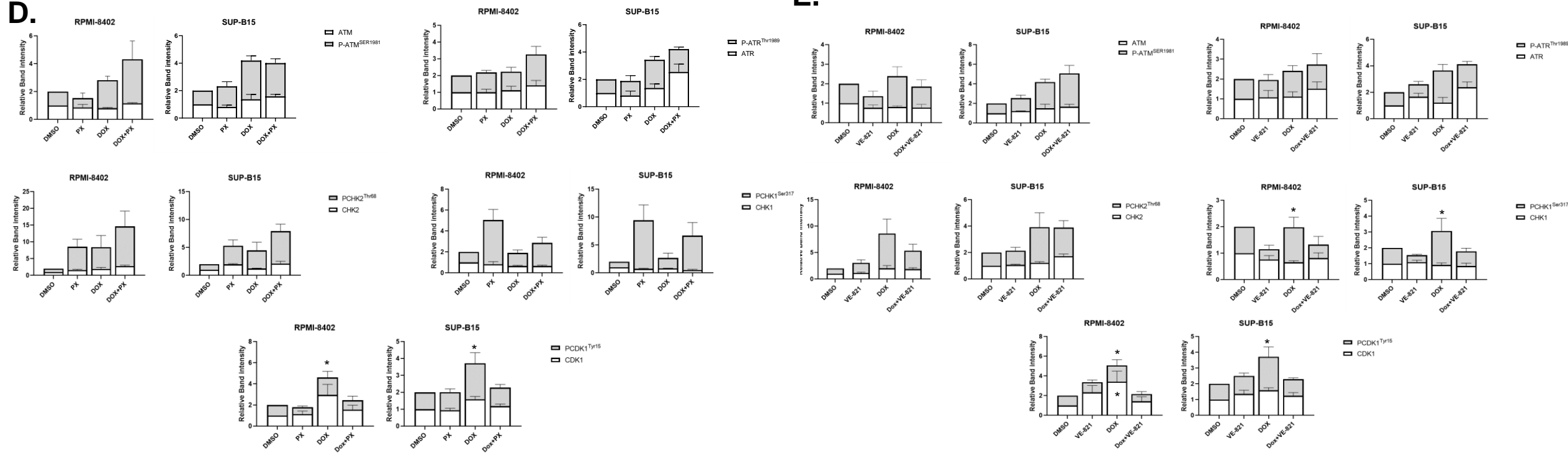

Supplement: Supplementary file 2 — Fig. S1: Effect of Dox in combination with VE-821 on cell viability in ALL cell lines and primary leukemic ALL cells. A) Reduction of cell viability of RPMI-8402, SUP-B15 and REH cells treated with Dox for 48 h and with VE-821 for further 24 h. Bars represent the mean ± standard deviation of at least three independent experiments. Statistical significance of the comparison between each drug concentration and DMSO treated cells was indicated by asterisks (*p < 0.05; **p < 0.01; p < 0.001). B) Cell viability analysis of primary leukemic ALL cells (n = 3) treated with Dox for 48 h and then with increasing concentrations of VE-821 for further 24 h or C) 48 h. Fig. S2: Effect of Dox in combination with PX on cell viability in ALL cell lines and primary leukemic ALL cells. A) Reduction of cell viability of RPMI-8402, SUP-B15 and REH cells treated with Dox for 48 h and with PX for further 24 h. Bars represent the mean ± standard deviation of at least three independent experiments. Statistical significance of the comparison between each drug concentration and DMSO treated cells was indicated by asterisks (*p < 0.05; **p < 0.01; p < 0.001). B) Cell viability analysis of primary leukemic ALL cells (n = 3) treated with Dox for 48 h and then with increasing concentrations of PX for further 24 h or C) 48 h. Fig. S4: Induction of tripolar spindles in ALL cell lines treated with Dox and VE-821. A) Immunofluorescence analysis of RPMI-8402, SUP-B15 and REH cells treated Dox (RPMI-8402, 0.1 μM; SUP-B15 and REH, 0.05 μM) for 48 h and then with VE-821 (5 μM) for further 3 h. In the figures, metaphases are showed by white circles and tripolar spindles are showed by yellow triangles; scale bars indicate 200 μm. Fig. S4: Induction of tripolar spindles in ALL cell lines treated with Dox and PX. A) Immunofluorescence analysis of RPMI-8402, SUP-B15 and REH cells treated Dox (RPMI-8402, 0.1 μM; SUP-B15 and REH, 0.05 μM) for 48 h and then with PX (0.1, 0.25 and 0.5 μM) for further 3 h. In the [file 10565_2021_9640_MOESM2_ESM.pdf]
